# A genetic screen identifies the E3 ubiquitin ligase MPSR1 as a suppressor of early flowering of the *sensitivity to red light reduced 1* mutant

**DOI:** 10.1038/s41598-025-26769-5

**Published:** 2025-11-21

**Authors:** Mikael Johansson, Wei Liu, Alexander Steffen, Morgan Vanderwall, Joshua M. Gendron, Dorothee Staiger

**Affiliations:** 1 RNA Biology and Molecular Physiology, Faculty of Biology, Bielefeld University, 33615 Bielefeld, Germany; 2 Department of Molecular, Cellular, and Developmental Biology, Yale University, New Haven, CT 06511 USA

**Keywords:** Ubiquitin ligase, Flowering, Arabidopsis, Circadian clock, SRR1, Full-genome sequencing, Plant development, Plant genetics, Plant physiology

## Abstract

The timing of floral transition is critical for plants to maximize reproductive success. In *Arabidopsis thaliana*, SENSITIVITY TO RED LIGHT REDUCED 1 (SRR1) delays the onset of flowering in non-favourable environmental conditions. Furthermore, SRR1 is required for the function of the circadian clock. In a genetic suppressor screen for mutants that alleviate early flowering of *srr1-1* mutants, we identified *ssm136* (*suppressor of srr1-1 mutant 136*) that represses *srr1-1* early flowering in short photoperiods. Bulk segregant analysis and full-genome sequencing identified *MISFOLDED PROTEIN SENSING RING E3 LIGASE 1* (*MPSR1*) as the causal gene. Outcrossing the *mpsr1-ssm* allele to wild-type showed that MPSR1 represses flowering independently of SRR1, but the effect is stronger in *srr1-1*, suggesting that MPSR1 and SRR1 act together in flowering time control. Co-immunoprecipitation showed that SRR1 and MPSR1 are able to physically interact in vivo. Expression analysis revealed that *MPSR1* is regulated by the circadian clock but not required for maintaining the clock itself. Furthermore, *MPSR1* expression is repressed by light and induced in short days, consistent with its role in floral repression under noninductive photoperiodic conditions.

**Supplementary Information:**

The online version contains supplementary material available at 10.1038/s41598-025-26769-5.

## Introduction

Proper timing of flowering is critical to balance sufficient vegetative development with maximized offspring and suitable seasonal conditions. For this, an intricate network of molecular signalling pathways has evolved in plants, to collect and interpret environmental and developmental signals, ensuring that flowering is initiated at the optimal time of the season and life cycle. Equally important is a layer of safeguards that prevents premature flowering in response to unfavourable conditions, both seasonal such as the light-dark cycle and temporary such as short-term abiotic or biotic stress. In the model plant *Arabidopsis thaliana* (Arabidopsis), it is well established that much of this regulation centres on the promotion or repression of the so-called “florigen” *FLOWERING LOCUS T (FT)*^1,2^.

The FT protein is synthesized in the leaves and subsequently moves through the vascular tissue to the shoot apex, where flowering is initiated. In the leaves, expression of *FT* is photoperiodically controlled via the CONSTANS (CO) protein, whose transcription is under circadian control via GIGANTEA (GI)^3,4^. Through light-coinciding interaction with FLAVIN BINDING, KELCH REPEAT, F-BOX1 (FKF1), FKF1 represses the activity of CDF transcription factors, which are repressors of *CO* and *FT* expression^5^. Thus, the *CO* transcript can accumulate, and CO protein is expressed in the afternoon in long days (LDs) and promotes *FT* expression by direct binding to its promoter.

In short days (SDs), flowering is repressed to avoid initiation of the reproductive process in non-favorable seasons. SENSITIVITY TO RED LIGHT REDUCED 1 (SRR1) ensures repression of flowering in non-inductive photoperiods in Arabidopsis. Accordingly, *srr1-1* mutant plants have a reduced ability to sense changes in day length, leading to particularly early flowering in SDs. By promoting the expression of direct repressors of *FT*, including the *CYCLING DOF FACTOR* (*CDF1)*, the *TEMPRANILLO* transcription factors and *FLOWERING LOCUS C* (*FLC)*, SRR1 represses flowering in non-inductive conditions^6^. Furthermore, in the *srr1-1* mutant genes regulated by the circadian clock as well as core clock genes including *CIRCADIAN CLOCK ASSOCIATED 1* (*CCA1*) with a morning peak and *TIMING OF CAB EXPRESSION 1* with an evening peak show short period oscillations with a reduced amplitude, whereas *SRR1* itself is not rhythmically expressed^7^. Additionally, SRR1 is involved in red light signaling, acting downstream of the red light photoreceptor phyB. The SRR1 protein lacks known domains that would hint to its molecular function. Its homologs have however been suggested to play a role in flowering time control in crop species. In *Brassica rapa* it has been associated with expression of *BrFT*^8^. In *Brassica napus*,* BnaSRR1* has been pinpointed as a candidate gene responsible for the morphotypic split between annual and biannual forms^9^. More recently, strong sub-functionalization of different *BnaSRR1* gene copies has been observed, suggesting an important role for *Bna.SRR1* beyond flowering in this species^10^.

To better understand how SRR1 acts in the molecular network of flowering time control, we performed ethylmethanesulfonate (EMS) mutagenesis of *srr1-1* plants and screened for novel mutants that alleviate the early-flowering phenotype. We previously described candidate suppressor mutants that promoted flowering in both SDs and LDs. By combining state-of-the-art whole genome re-sequencing and traditional mapping, we identified several candidates acting in the same genetic pathway as SRR1, among them HAD-FAMILY REGULATOR OF DEVELOPMENT AND FLOWERING 1 (HDF1). HDF1 is the first haloacid dehalogenase implicated in flowering time control in Arabidopsis and likely regulates *FLC.* The domain structure of HDF1 differs slightly from that of known consensus haloacid dehalogenases and thus its substrates remain to be identified^11^.

Among candidates that specifically acted in SDs, one of the affected genes mapped to a locus encoding an E3 ubiquitin ligase. Ubiquitin-mediated proteolysis is an important regulatory tool in many light-related signalling pathways^12^. In particular, this mechanism helps to overcome stresses such as extreme hot and cold temperatures, salinity, drought and infection of pathogenic microorganisms. These circumstances disturb cellular homeostasis, in which the plant makes use of the ubiquitin proteasome system (UPS) in order to maintain basal functions. E3 ubiquitin ligases play a pivotal role in the function of the UPS. E1 activating enzymes are responsible for the activation of ubiquitin, which is subsequently transferred to an E2 conjugating enzyme and finally to the substrate by a specific E3 ubiquitin ligase. The ubiquitinylated protein is finally targeted for degradation by the 26 S proteasome^13^.

E3 ligases are classified into HECT (HOMOLOGY TO E6-AP C-TERMINUS)-type, RING (REALLY INTERESTING NEW GENE)-type, U-box-type, and multi-complex E3 ligases, based on their functional domains^13^. RING-type E3 ligases possess a RING motif that coordinates two zinc ions, creating a docking place for E2 conjugating enzymes^14^. HIGH EXPRESSION OF OSMOTICALLY RESPONSIVE GENES 1 (HOS1) and CONSTITUTIVELY PHOTOMORPHOGENIC 1 (COP1), both RING E3 ligases, have been shown to play crucial roles in the plant’s response to stress and flowering. HOS1 is important in modulating responses to extreme cold^15^ while COP1 has been shown essential for etiolation by acting as a repressor of photomorphogenesis^16^. COP1 also works in concert with SUPPRESSOR OF PHYA-105 (SPA) proteins to mediate the degradation of CO and repress photoperiodic flowering^17^. *hos1* mutants have an early-flowering phenotype, as if constitutively vernalized, which is consistent with low expression levels of *FLC*^18^. This, combined with HOS1 preventing increased CO levels in SDs and *FT* expression in early hours of the day, could be a link between controlling cold stress responses and flowering time^19^. The cross-signalling between other kinds of abiotic stress and the timing of flowering is however largely unexplored.

Here we show that our genetic screen of second-site suppressor mutants of *srr1-1* resulted in the *ssm136* mutant that partially rescued the early-flowering phenotype of *srr1* and counteracted the increased level of the floral integrator gene *FT* in *srr1*. Mapping by sequencing revealed that the causal mutation localizes to a gene encoding MISFOLDED PROTEIN SENSING RING E3 LIGASE 1 (MPSR1). MPSR1, also known as SNIPER2, is involved in protein quality control under proteotoxic stress and targets nucleotide-binding leucine-rich repeat (NLR) immune receptors for degradation along with its homolog SNIPER1^20,21^. For the first time it is now implicated as a potential genetic interactor of SRR1, specifically affecting flowering time in SD conditions.

As SRR1 has an impact on the circadian clock but itself is not rhythmically expressed, we characterized the *MPSR1* expression pattern throughout the day. Publicly available microarray data indicated that the *MPSR1* transcript undergoes circadian oscillations. An *MPSR1* promoter-*LUCIFERASE* reporter revealed that *MPSR1* promoter activity is regulated by the circadian clock. Moreover, MPSR1 is induced in short photoperiods. As no defect in circadian rhythmicity was detected when tracking the *LUCIFERASE* reporter driven by the promoter of the circadian clock component *CIRCADIAN CLOCK ASSOCIATED 1* in the *mpsr1* mutant, MPSR1 is not essential to maintain the period of the circadian clock in contrast to what is observed for SRR1^7^.

## Results

### A *srr1* suppressor screen identifies candidates with short day specific flowering

SRR1 is an important regulator of flowering time, indirectly repressing expression of *FT* under noninductive conditions^6^. As it has no known domains that would suggest a particular function for the protein, a second site suppressor screen was performed for novel genes regulating flowering in concert with SRR1. Flowering time was monitored in EMS-mutagenized *srr1-1* loss-of-function mutants in SDs at 16°C where *srr1-1* shows the strongest phenotype^6,22^.

**Fig. 1:**
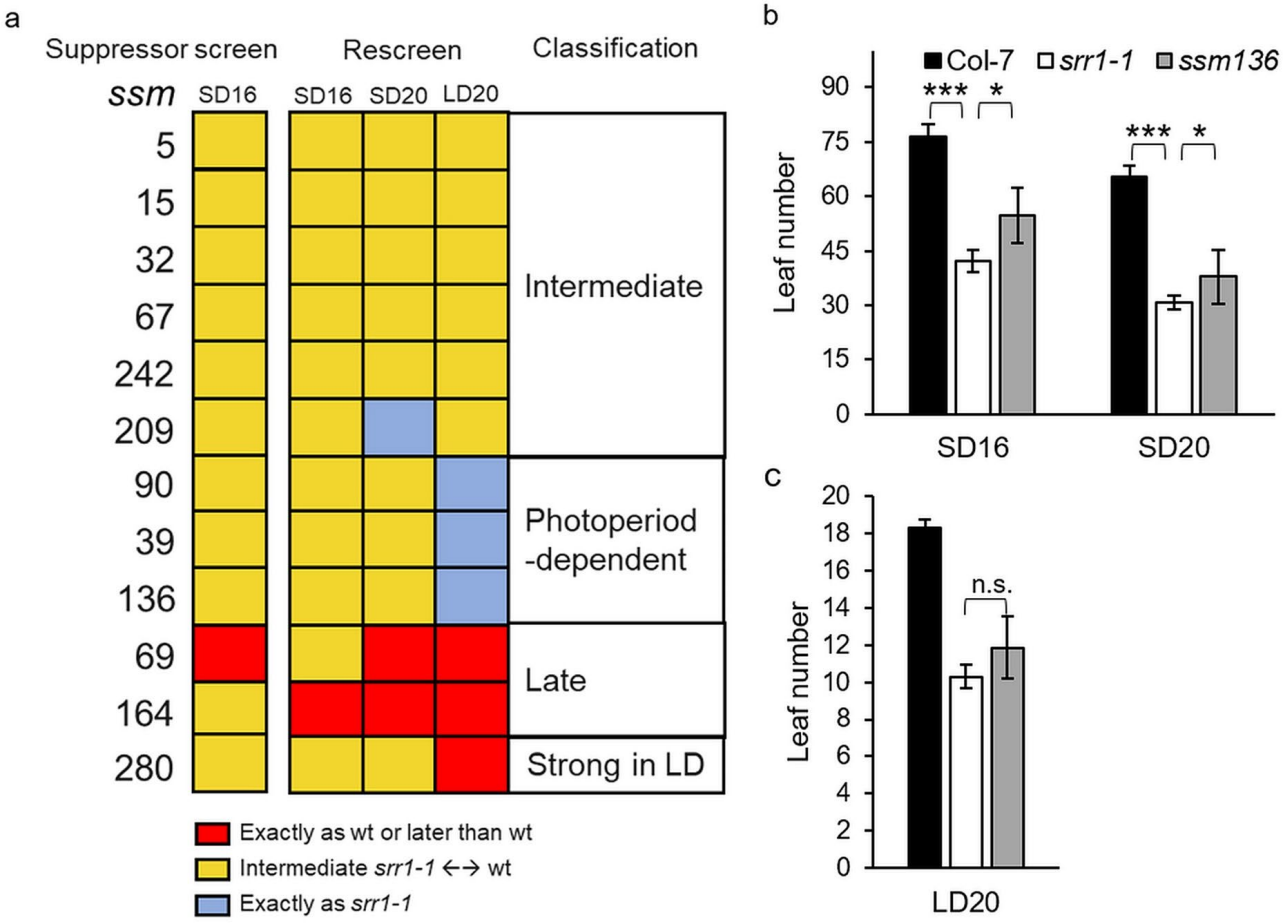
Flowering time of *suppressor of srr1-1* (*ssm*) candidate mutants. (**a**) Summary of the flowering time of candidate *ssm* mutants in SDs at 16°C and re-screen of candidate *ssm* mutants in SDs at 16°C, 20°C and in LDs at 20°C. The number of leaves was counted for 10–12 plants each. *Ssm* mutants with delayed flowering also in LDs, partially rescuing the early flowering of *srr1-1* were classified as “*intermediate*” candidates (*ssm5*, *ssm15*, *ssm67* [*also designated hdf1*^*12*^], *ssm242*, and *ssm209*) The suppressive effect of s*sm90*,* ssm39*, and *ssm136* is SD-specific and they were classified as “*photoperiod-dependent*”. *Ssm69* and *ssm164* bolted with very high leaf numbers in both 20°C SD and LD conditions and were classified as “*Late*”. The *ssm280* flowered much later than wt in LDs at 20°C, while displaying an intermediate phenotype in SDs. (**b**, **c**) Flowering time of *ssm136*, *srr1-1* and Col-7 was measured in SDs at 16°C and 20°C (**b**) and in LDs at 20°C (**c**). The number of leaves was counted for 10–15 plants each and shown as mean ± SD. A Kruskal-Wallis test was performed to show statistical significance (*p < 0.05, ***p < 0.001, n.s. not significant).

Specifically, 1100 mutagenized seeds from 11 different batches treated with EMS were screened for candidates flowering with a higher number of leaves at bolting than *srr1-1*. In the initial round of screening, 12 *suppressor of srr1-1 mutant* (*ssm*) candidates were identified that bolted significantly later than *srr1-1* and produced viable seeds for further experiments. The candidates were further classified in different rounds of screening that we have recently described in greater detail^11^. Three mutants, *ssm90*,* ssm39*, and *ssm136* did not show any difference in leaf number at bolting compared to *srr1-1* in LDs at 20°C, suggesting that the suppressive effect on flowering is SD-specific (Fig. 1a). Of these photoperiod-dependent suppressor mutants, *ssm136* showed a reproducible SD-specific flowering phenotype in the re-screening process and displayed healthy growth in general, suggesting a low number of background mutations that may influence further analysis. Therefore, it was singled out for further characterization.

### *ssm136* shows a delay in flowering only under short day conditions

The *ssm136* mutant flowered later than *srr1-1* in the screen in SDs at 16°C. Plants in the M3 generation were tested for their flowering behavior in the same growth conditions to confirm the mutant phenotype. This revealed a delay in flowering represented by an increase of 15 leaves at time of bolting compared to *srr1-1* (Fig. 1b). In SDs at 20°C a delay in flowering of similar proportion compared to *srr1-1* was observed, although leaf numbers were lower for all genotypes, resulting in later flowering than *srr1-1*, but still earlier than the wt control plants (Fig. 1b). In LDs at 20°C, however, *ssm136* plants bolted with a similar leaf number as *srr1-1*, suggesting that the locus mutated in *ssm136* is of particular importance in SD conditions (Fig. 1c). In addition, the *ssm136* plants flowered with a lower leaf number at 20°C compared to 16°C, as observed in Col wt plants. Thus, the ambient temperature response was maintained in SDs in *ssm136*.

### A point mutation in E3 ubiquitin ligase *MPSR1* is causal for the *ssm136* phenotype

The identification of the causal locus of the *ssm136* suppressor mutant was performed by a full genome re-sequencing approach using bulk segregant analysis^23^. F2 segregating plants of a backcross performed between *srr1-1* and *ssm136* (BC1F2) were grown in SDs at 16°C. To confidently select plants carrying the causal mutation, individuals with at least 25% more leaves at bolting than the *srr1-1* control population were sampled for leaf material. Samples from 29 plants were pooled and total DNA was extracted. The DNA pool was subsequently sequenced, with a coverage of ca. 50-fold. Simultaneously, a pool of 10 *srr1-1* plants in the Col-7 background was sampled and sequenced as a reference, since Col-7 is not identical to the Col-0 ecotype used as a reference genome^24^.

**Fig. 2:**
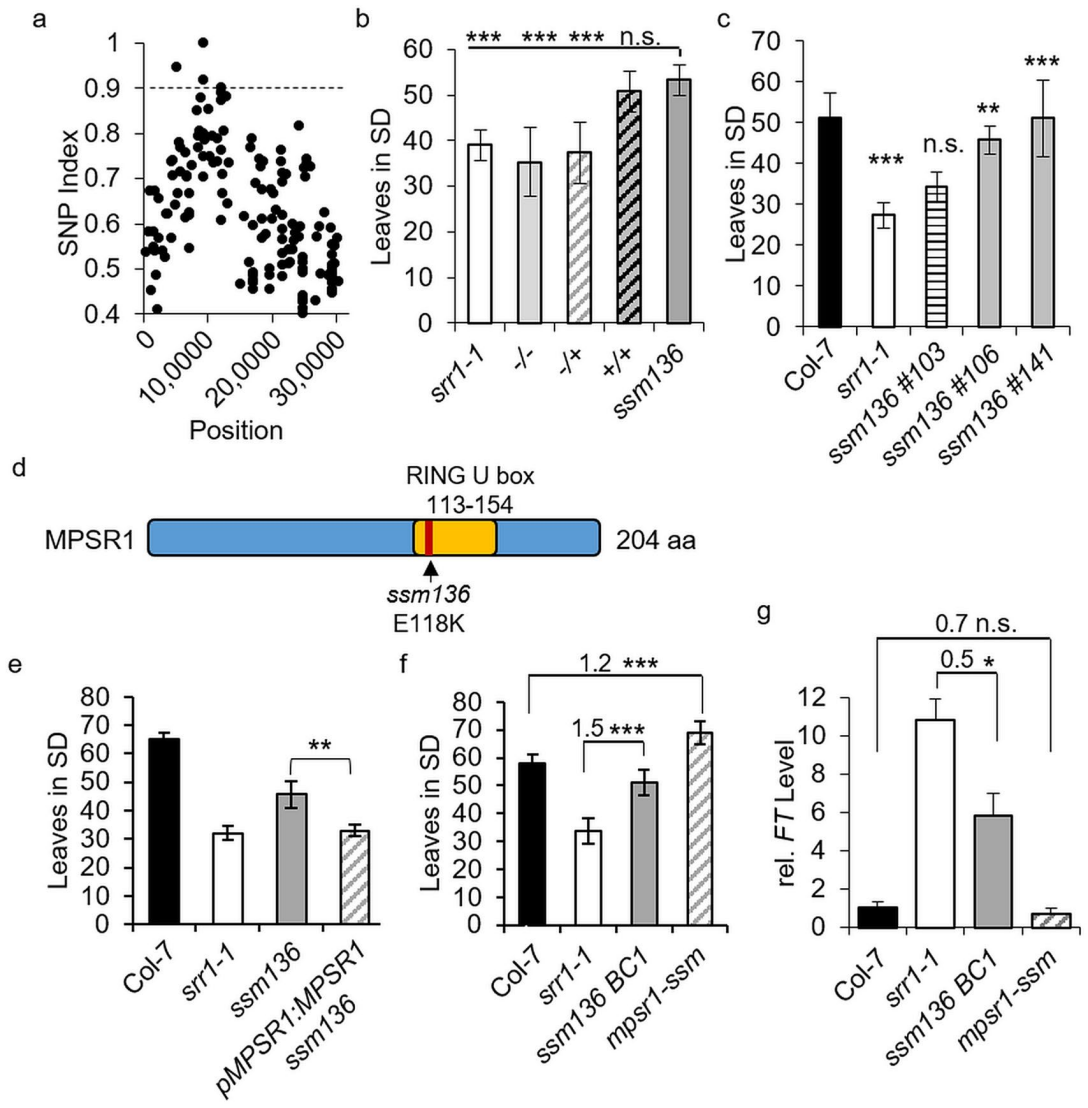
Identification of the causal mutation in *ssm136*. (**a**) SNP index for chromosome 1. Only candidates with a SNP index greater 0.9 were selected for further investigation. (**b**) Flowering time of the segregating *ssm136* x *srr1-1* BC1F2 population. Plants from *srr1-1*, *ssm136* and plants either homozygous (+/+), heterozygous (+/–) or wt (–/–) for the SNP in At1g26800 were grown in SDs (*n* = 10–12). (**c**) Flowering time of an independently segregating *ssm136* population. Col wt, *srr1-1*, *ssm136* #103 without SNP in At1g26800, *ssm136* #106 and *ssm136* #141 with SNP in At1g26800 were grown in SDs (*n* = 10–16). (**d**) Scheme of MPSR1 protein. The position of the E to K mutation caused by the SNP is indicated. (**e**) Flowering time of the *ssm136* mutant complemented with genomic *MPSR1* construct (*n* = 12–15). All flowering time experiments were performed in SDs and leaf number was scored upon bolting. Data is shown as mean ± SD. A Kruskal-Wallis test was performed to show statistical significance (**p < 0.01, ***p < 0.001, n.s. not significant). (**f**) Flowering time of the *mpsr1-ssm* mutant compared to *ssm136*. Col wt, *srr1-1*, *ssm136* and *mpsr1-ssm* were grown in SDs. Leaf number was scored upon bolting from 15 plants each and is shown as mean ± SD. The ratio of delay between Col and *mpsr1-ssm and srr1-1* and *ssm136* was calculated and is given above the bar chart. (**g**) *FT* levels in Col-7 wt, *srr1-1*, *ssm136* and *mpsr1-ssm* grown in SDs for 30 days. qRT-PCR data of 3 biological replicates normalized to *PP2A*.

The sequencing revealed 12,478 single nucleotide polymorphisms (SNPs) between the TAIR10 Col-0 reference genome and *ssm136* mutants. Of these, 1,496 SNPs were unique between *srr1-1* and *ssm136* (Supplementary Table S1 online). After intergenic SNPs were filtered out, a SNP Index was calculated for the remaining 596 SNPs and plotted according to the position on each chromosome (Supplementary Fig. S1 online). This revealed a clear peak of SNP enrichment on chromosome I (Fig. 2a). The three SNP candidates with the highest SNP Index (above 0.9) in this region were selected for further confirmation (Table 1). The presence of the SNPs in the population of plants that was used to create the sequencing pool was confirmed using specifically designed dCAPS markers (Supplementary Table S2 online).

**Table 1:** *ssm136* candidate loci with the highest SNP index.

Locus	SNP Index	Position	Nucleotide change	Type	Region
AT1G27080	1	9402953	C895T	Nonsynonymous SNP	Exonic
AT1G14830	0.95	5107921	G1717A	Nonsynonymous SNP	Exonic
AT1G26800	0.92	9285839	G352A	Nonsynonymous SNP	Exonic

To elucidate which of the three SNPs was the causal mutation, a segregating *ssm136 x srr1-1* BC1F2 population that was independent from the population of the sequencing pool was grown in SDs at 16°C. Subsequently, all plants in the population were tested for the presence of the different SNPs using the dCAPS markers. This was correlated with the flowering time of the individual plants. Only plants homozygous for the SNP located in At1g26800 flowered later than *srr1-1* and with a similar leaf number at bolting as the *ssm136* mutant, strongly suggesting that this was the causal mutation. Plants without the SNP or plants heterozygous for the SNP flowered with about the same leaf number as *srr1-1*. Taken together, the SNP segregates as a recessive trait, likely indicating a loss-of-function mutation (Fig. 2b). The At1g26800 SNP Index of 0.92 suggested the presence of a small proportion of non-mutated material in the sequencing pool, which could be explained by one plant in the pool not carrying this SNP (*ssm136 #103*), when tested with the specific dCAPS marker. This was taken advantage of as an internal control and seeds were harvested from this plant and two other independent members of the pool carrying the *ssm136* mutation (*ssm136 #106* and *ssm136 #141*). These were subsequently used for an additional flowering time experiment which revealed that *ssm136 #103* without the SNP flowered as early as *srr1-1*, while *ssm136 #106* and *ssm136 #141* with the SNP had a significantly delayed flowering compared to *srr1-1*, further supporting that the presence of the SNP in At1g26800 is critical for the *ssm136* phenotype (Fig. 2c). At1g26800 encodes MPSR1, a RING-type E3 ligase recently shown to be involved in protein quality control under proteotoxic stress^20^. Its RING domain is necessary for the E3 ligase activity. The G to A point mutation in *ssm136*, at base number 352 in the genomic sequence of MPSR1 leads to an E to K change in amino acid 118, within the well conserved RING domain of the protein (amino acids 113–154) (Fig. 2d). Introduction of a genomic copy including the promoter of MPSR1 (*pMPSR1:MPSR1*) into *ssm136* complemented the late flowering phenotype, confirming that this was the causal locus (Fig. 2e).

To see whether the E to K change is unique or may naturally appear in other ecotypes, the MPSR1 amino acid sequence was examined using the 1001proteomes non-synonymous SNP browser^25^. However, the *ssm136* E to K change is not present in any ecotype included in the database, suggesting that E118 is well conserved and critical for MPSR1 function.

### MPSR1 plays a role in flowering time control

MPSR1 has recently been shown to function in protein quality control under proteotoxic stress and plant immune responses and MPSR1 overexpressing plants are late flowering^20,26^, but its precise role in control of flowering is hitherto unknown. To examine this, a *mpsr1-ssm* single mutant was created by outcrossing the *srr1-1* allele and named as such due to its origin and in line with the terminology used for previously identified *mpsr1* mutants^20^. A phenotypic comparison of *mpsr1-ssm*, *ssm136*, *srr1-1* and Col-7 wt plants revealed that *mpsr1-ssm* resembles wt, although plants appear slightly smaller with rounded leaf blades. The *ssm136* mutant reverts the dwarf phenotype of *srr1-1* but still shows elongated petioles comparable to *srr1-1*. This indicates again that *ssm136* is a partial suppressor of SRR1-dependent development (Supplementary Fig. S2 online).

The *mpsr1-ssm* mutant was then tested for flowering time in SDs, under which conditions *ssm136* delays flowering in the *srr1-1* background. This revealed a moderate, but statistically significant (Student’s t-test, *p* > 0.01) delay of flowering in the *mpsr1-ssm* plants compared to wt, showing that this mutant does have an independent effect on flowering, but that the effect is strongly enhanced when SRR1 is not present (Fig. 2f). In accordance to the flowering time phenotype, expression of the floral activator *FT* was slightly but not significantly decreased in *mpsr1-ssm* compared with Col-7 (Fig. 2g). Again, the effect was stronger in the *srr1-1* background, where *ssm136* significantly reduced the *FT* level to around 50% of the level observed in *srr1-1.*

**Fig. 3:**
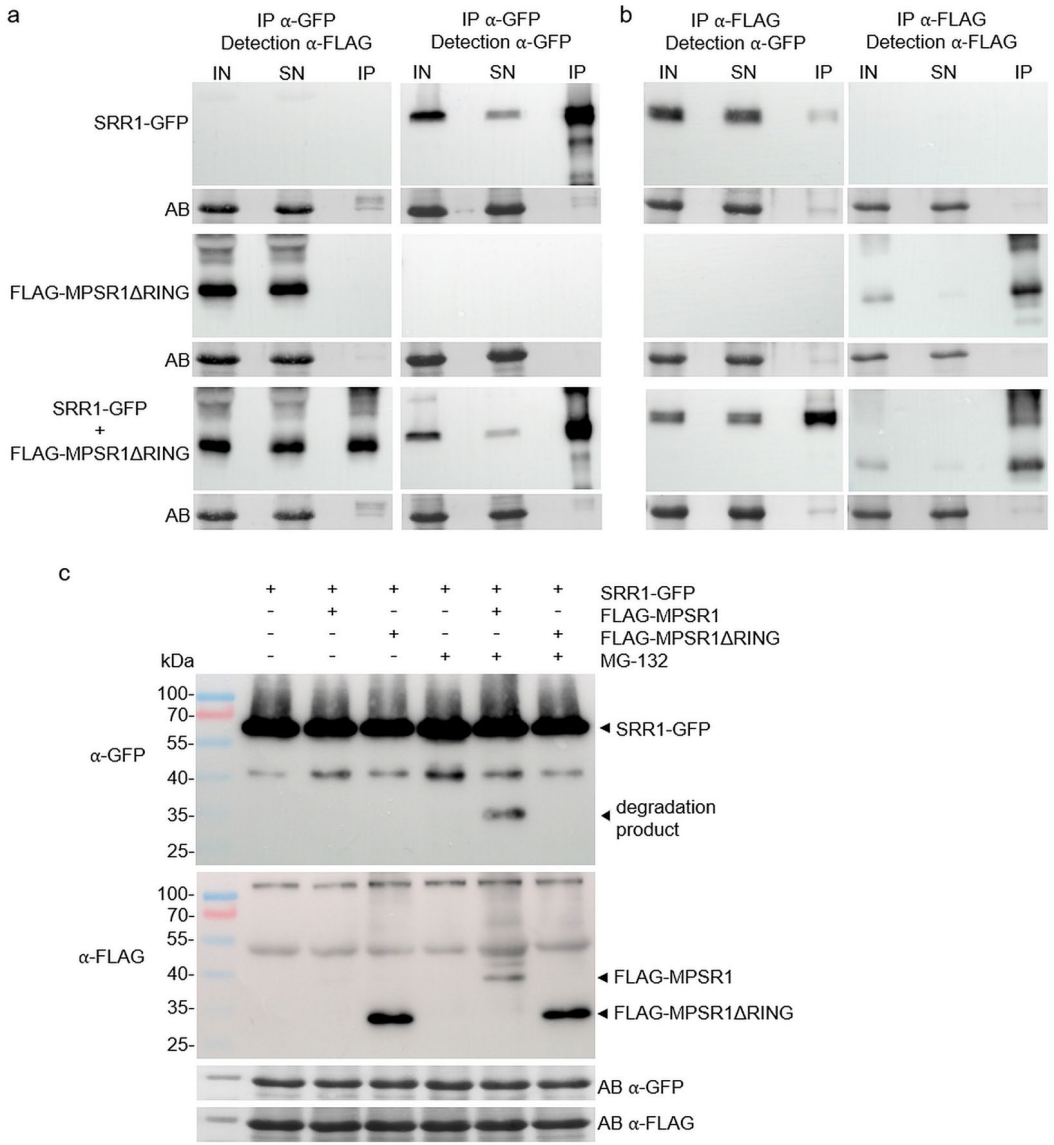
Co-Immunoprecipitation of SRR1 and MPSR1. (**a**) Agrobacteria carrying 35S:SRR1-GFP and 35S:FLAG-MPSR1ΔRING were each infiltrated separately and co-infiltrated into *N. benthamiana* leaves and harvested after 2 days. Native protein extracts were subjected to IP with GFP-Trap beads. 1% of input (IN), 1% of supernatant (SN) and 66% of the beads (IP) were loaded onto SDS-gels and detected with an α-FLAG antibody to show coprecipitation of FLAG-MPSR1ΔRING. For detection of successful pulldown of SRR1-GFP on a separate gel, 1% of IN and SN and the remaining 33% of beads were loaded and the blot was detected with α-GFP antibody. (**b**) Reverse Co-IP of 35 S: FLAG-MPSR1ΔRING and 35 S: SRR1-GFP. FLAG-MPSR1ΔRING was immunoprecipitated with α-FLAG beads and co-precipitated SRR1-GFP was detected with the α-GFP antibody. To show successful pulldown of FLAG-MPSR1ΔRING, the same membrane was incubated with α-FLAG antibody after stripping of the α-GFP antibody. Amidoblack (AB) staining of the membranes served as loading control. The uncropped blots are shown in Supplementary Fig. S6 online. (**c**) In vivo degradation assay of SRR1-GFP. Agrobacteria carrying 35 S: FLAG-MPSR1 or 35 S: FLAG-MPSR1ΔRING and 35 S: SRR1-GFP were co-infiltrated into *N. benthamiana* leaves. After 1 day, half of the plants were infiltrated with 50 µM MG-132 in DMSO while the other half was infiltrated with DMSO alone as mock control. Leaves were harvested after 2 days. 30 µg of denaturing protein extracts were loaded on SDS-gels and detected with α-GFP and α-FLAG antibodies, respectively. Amidoblack (AB) staining of the membranes served as loading control. The uncropped blots are shown in Supplementary Fig. S7 online.

To test whether the interplay between SRR1 and MPSR1 in flowering control was due to a direct interaction of SRR1 and the E3 ligase MPSR1, we performed co-immunoprecipitation (Co-IP) of transiently expressed SRR1-GFP and 3xFLAG-tagged MPSR1 (FLAG-MPSR1) in *N. benthamiana*. While a test expression showed that SRR1-GFP was readily expressed at high levels, no signal could be detected for FLAG-MPSR1, likely owing to self-ubiquitination and further proteasomal degradation. We therefore deleted the RING domain to omit E3 ligase activity (FLAG-MPSR1ΔRING) and found that this variant readily expresses in *N. benthamiana* (Supplementary Fig. S3 online). After co-infiltration of agrobacteria carrying SRR1-GFP or FLAG-MPSR1ΔRING, a pull-down with GFP Trap beads and a subsequent detection with the α-FLAG antibody showed that MPSR1ΔRING co-precipitates with SRR1-GFP, suggesting that both proteins physically interact and that the interaction does not require the RING domain (Fig. 3a). As a control we performed the reciprocal Co-IP, pulling down FLAG-MPSR1ΔRING with anti-FLAG beads. Immunoblot analysis with the α-GFP antibody revealed co-precipitation of SRR1-GFP (Fig. 3b). In contrast, a mock Co-IP with GFP alone and FLAG-MPSR1ΔRING detected no MPSR1ΔRING in the immunoprecipitated fraction, ruling out an unspecific interaction with the GFP moiety (Supplementary Fig. S4 online). Thus, SRR1 is able to interact in vivo with MPSR1ΔRING.

To further explore if MPSR1 might be an E3 ligase responsible for SRR1 ubiquitination and subsequent degradation, we performed an in vivo degradation assay. SRR1-GFP was expressed in *N. benthamiana* together with either FLAG-MPSR1 or FLAG-MPSR1ΔRING. After 1 day of co-expression, half of the plants were infiltrated with 50 µM of the proteasome inhibitor MG-132 while the other half served as mock control. Samples were harvested after an additional 12 h and the appearance of degradation products was monitored with the α-GFP antibody. All samples expressed the full-length SRR1-GFP and showed a degradation product with lower molecular weight (MW) around 40 kDa. No additional degradation products were detected in mock treated samples. However, in plants treated with MG-132, a prominent additional degradation product appeared at a MW of 35 kDa in the sample co-expressing FLAG-MPSR1 with SRR1-GFP. In the sample co-expressing FLAG-MPSR1ΔRING, this additional degradation product was absent (Fig. 3c). As observed before, no full-length FLAG-MPSR1 protein could be detected in mock treated plants while FLAG-MPSR1ΔRING was expressed at considerable levels. However, in MG-132 treated plants a weak band corresponding to full-length FLAG-MPSR1 appeared, indicating that MPSR1 auto-regulation via self-ubiquitination and subsequent degradation was at least partially inhibited and that formation of the 35 kDa degradation product depended on detectable amounts of full-length FLAG-MPSR1 in the presence of MG-132. This shows that MPSR1 is potentially able to target SRR1 for degradation through the 26 S proteasome.

As we initially infiltrated excess amounts of agrobacteria carrying SRR1-GFP to foster the detection of low abundant degradation products, we went on with a 5-fold lower dosage of SRR1-GFP to see whether MPSR1 was able to reduce the amount of SRR1-GFP protein or whether SRR1-GFP accumulates in MG-132 treated samples. However, we could not detect changes in SRR1-GFP levels upon FLAG-MPSR1 co-infiltration or MG-132 treatment. Instead, the level of SRR1-GFP remained relatively stable across the whole experiment with slight variations likely owing to the transient expression system. Notably, upon longer exposure of the blot, the 35 kDa degradation product again appeared upon co-expression of FLAG-MPSR1 with SRR1-GFP under MG-132 treatment (Supplementary Fig. S5a online). To test the specificity of the substrate, we performed the degradation assay with co-expression of MPSR1 and GFP only. No degradation products were detected in MG-132 treated samples (Supplementary Fig. S5b online). In conclusion, this shows that SRR1 is a potential substrate of MPSR1, though indirect regulation, e.g. through related E3 ligases present in the *N. benthamiana* system, cannot be fully ruled out.

### *MPSR1* expression is under control of the circadian clock

**Fig. 4:**
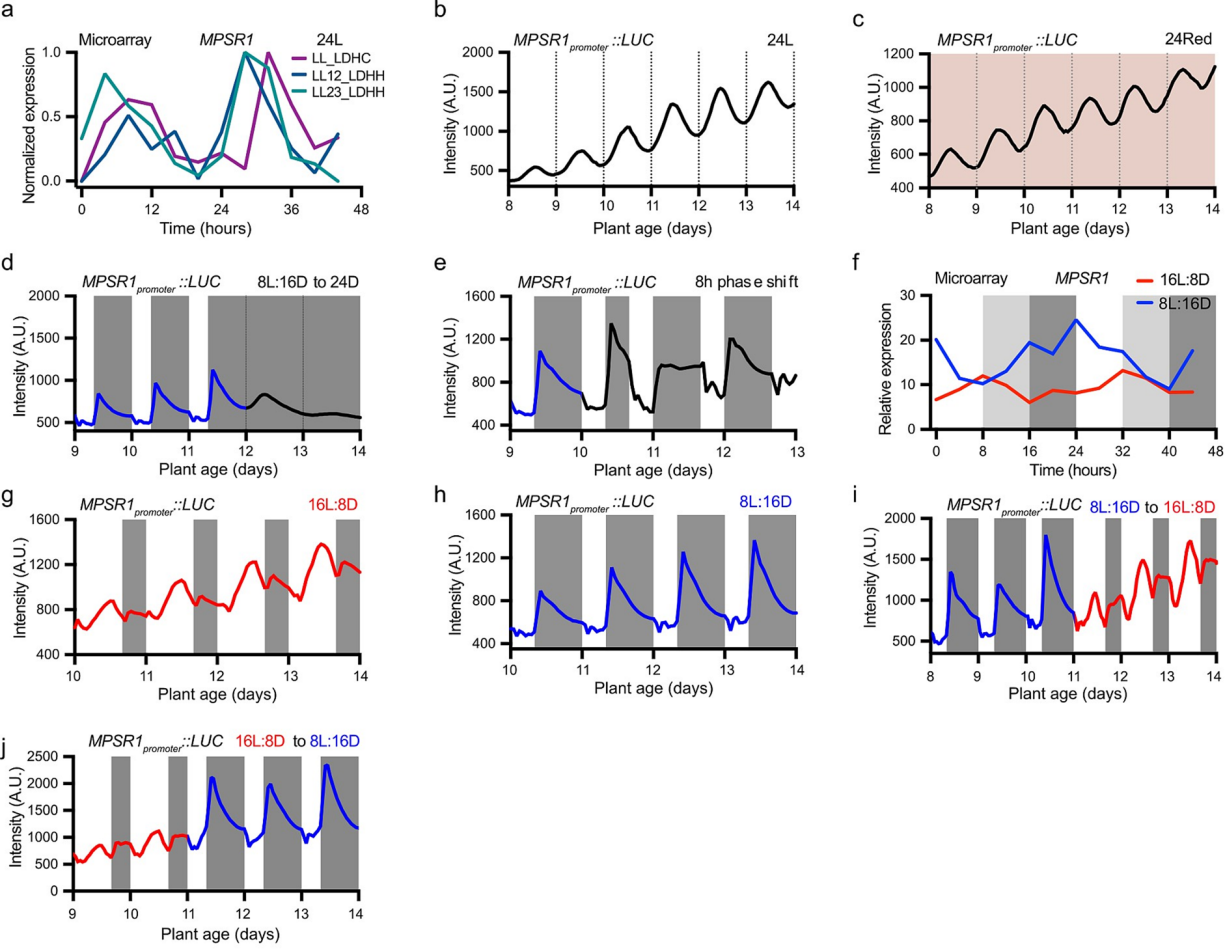
*MPSR1* is highly induced in short days. (**a**) Normalized expression pattern of *MPSR1* in continuous white light (24 L) from the three microarray datasets, LL_LDHC, LL12_LDHH, LL23_LDHH. (**b**–**e**) Traces of *MPSR1*_*promoter*_::*LUC* expression from plants grown in constant white light (24L) (**b**), constant red light (24Red) (**c**), 8L:16D to 24D (**d**), 8L:16D with 8 h dawn phase shift at day 10 (**e**). Grey shading represents dark periods. Lines represent the intensity of traces. (**f**) Expression of *MPSR1* in 16L:8D and 8L:16D in microarray datasets. (**g**–**j**) Traces of *MPSR1*_*promoter*_::*LUC* expression from plants grown in 16L:8D (**g**), 8L:16D (**h**), 8L:16D and shifted to 8L:16D (**i**), 16L:8D and shifted to 8L:16D (**j**).

Given that SSR1 influences circadian oscillations of core clock and clock output genes, we explored the potential involvement of MPSR1 in circadian clock function. First, we analysed *MPSR1* transcript levels across three publicly available microarray datasets under constant white light (LL) conditions: LL_LDHC, LL12_LDHH, and LL23_LDHH (Fig. 4a)^27^. *MPSR1* exhibits rhythmic expression under continuous light, with a peak between Zeitgeber time 6 (ZT6, 6 h after lights on) to ZT10 (correlation of 0.85 in LL_LDHC; 0.8 in LL12_LDHH; 0.91 in LL23_LDHH indicating rhythmicity).

To precisely monitor *MPSR1* expression, we generated a transgenic line expressing the *Luciferase* gene under the control of *MPSR1* promoter and measured the luminescence in a circadian time course. The reporter line was initially grown under 12 h light:12 h dark (12L:12D) conditions before being shifted to constant light (Fig. 4b). The expression pattern observed in this experiment was consistent with those seen in the microarray datasets (Fig. 4a). *MPSR1* expression was rhythmic, with a relative amplitude error (RAE) of 0.31, a period of 24.05 h and a phase of ZT9.9, as calculated by the Biodare2 platform (biodare2.ed.ac.uk)^28,29^. This rhythmic expression pattern was also observed under constant red mono-spectral light condition (RAE = 0.24, period = 23.66, phase = 9.38) (Fig. 4c). The circadian clock dampens quickly in constant darkness, prompting us to test *MPSR1* expression in constant darkness. We initially grew the plants in 8L:16D to entrain the clock before shifting them to continuous darkness (Fig. 4d). In 8L:16D, *MPSR1* exhibited rhythmic expression, with a rapid induction after dusk. After the shift to continuous darkness, *MPSR1* rhythmicity was dampened within 48 h, suggesting that its rhythmic activation relies on the circadian clock.

Next, we conducted a phase shift experiment by growing the plants in 8L:16D condition and advancing the phase of dawn by 8 h on day 10 (Fig. 4e). Circadian clock-regulated genes typically require time to re-entrain to a new dawn following a phase shift. On the first day after the phase shift, we observed a low amplitude and extended phase of *MPSR1* expression pattern, but by the second day after the phase shift, *MPSR1* expression had re-entrained, returning to its normal phase. Taken together, those results indicate that the circadian clock controls the expression of *MPSR1*, with a phase around the middle of the day.

### *MPSR1* is induced in short day growth conditions

The expression of *MPSR1* is regulated by the circadian clock and MPSR1 is required for the impact of SRR1 on the transition to flowering in SDs. This led us to investigate *MPSR1* expression under different photoperiod light regimes. We first monitored *MPSR1* transcript levels under 8L:16D and 16L:8D conditions using microarrays datasets (Fig. 4f). By calculating the relative daily expression integral (rDEI = sum of 24-hour expression in 8L:16D / sum of 24-hour expression in 16L:8D), we found that *MPSR1* expression was significantly higher in 8L:16D as compared to 16L:8D, with an rDEI_8L:16D/16L:8D_ value of 1.71^30^.

We then monitored expression of the *MPSR1*_*promoter*_::*LUC* transgenic reporter line. In 16L:8D, *MPSR1* promoter activity phased towards the end of the light period, with a slight rise again after dusk (Fig. 4g), consistent with the pattern observed for *MPSR1* expression in microarrays (Fig. 4f). In 8L:16D, *MPSR1* promoter activity was low during the day but rapidly increased after dusk, followed by a gradual decrease throughout the night, suggesting that *MPSR1* promoter activity is light-repressed (Fig. 4h). As it is difficult to compare amplitudes from plants grown in separate photoperiod conditions, we performed photoperiod flip experiments, switching from 8L:16D to 16L:8D and vice versa (Fig. 4i, j). Two main observations were noted in these experiments. First, the expression patterns adjusted immediately to the new light regime. Second, the amplitude of expression is clearly lower in 16L:8D than 8L:16D. Collectively, the microarray data and *MPSR1*_*promoter*_::*LUC* reporter assay suggest that *MPSR1* expression is induced in SDs as compared to LDs.

### MPSR1 is not required for maintaining the period of the circadian clock

**Fig. 5:**
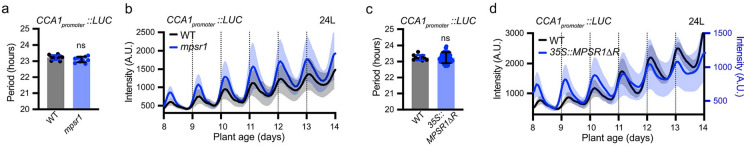
*MPSR1* is not required for maintaining the period of the circadian clock. (**a**) Period of *CCA1*_*promoter*_::*LUC* in the wild type and *mpsr1* mutant background. Error bars indicate SD (*n* = 10; ns, not significant (Welch’s t-test)). (**b**) Traces of *CCA1*_*promoter*_::*LUC* in the wild type and *mpsr1* mutant background. Shadings indicate SD (*n* = 10). (**c**) Period of *CCA1*_*promoter*_::*LUC* in wild type and *MPSR1* decoy (*35S::MPSR1ΔR*) transgenic lines. Wild type plants are identical to a and b. Error bars indicate SD (*n* = 10 in WT and *n* = 100 in *MPSR1* decoy), ns, not significant (Welch’s t-test). (**d**) Traces of *CCA1*_*promoter*_::*LUC* in wild type and *MPSR1* decoy (*35S::MPSR1ΔR*) transgenic lines. Shadings indicate SD (*n* = 10 in wild type and *n* = 100 in *MPSR1* decoy).

Circadian clock mutants often exhibit flowering time defects, like those seen in the *mpsr1* mutant, as they cannot discriminate inductive long photoperiods from non-inductive short photoperiods. Because SRR1 affects flowering time and clock function, we sought to determine if MPSR1 can also affect clock function. To determine whether MPSR1 influences the circadian timing system, we introduced the *CCA1*_*promoter*_::*LUC* reporter to the *mpsr1* mutant background (Fig. 5a, b) or to plants expressing the *MPSR1* decoy (*35S::MPSR1∆RING*) (Fig. 5c, d), and monitored LUC activity under constant light conditions (24 L)^31,32^. We observed no difference in the period of *CCA1* expression between the *mpsr1* mutant and the wild type (Fig. 5a, b). This conclusion was further supported by the absence of period defects when overexpressing *MPSR1ΔRING* (*35S::MPSR1*∆*R*) in the *CCA1*_*promoter*_::*LUC* reporter line (Fig. 5c, d). These results suggest that MPSR1 is not essential for maintaining the period of the clock.

## Discussion

SRR1 has an important role in preventing premature flowering under noninductive conditions. Here, we performed a suppressor screen of the early-flowering *srr1-1* mutant to find further molecular components acting in the same genetic pathways. This resulted in several mutant candidates that partially rescued the early-flowering phenotype of *srr1-1*. One of these candidates, *ssm136* that was classified as photoperiod-dependent, was further characterized. The *ssm136* mutant displayed a delayed flowering specifically under SD conditions (Fig. 1). Mapping-by-sequencing revealed that the causal mutation was located on chromosome 1 and its identity could be confirmed via dCAPS analysis and complementation, revealing that the mutated locus codes for the E3 ligase MPSR1. Further analysis showed that the resulting E to K nonsynonymous amino acid change leads to a delayed flowering also in lines where the *srr1-1* mutation had been outcrossed. A previous study had shown that MPSR1/SNIPER2 and SNIPER1 act together on a broad set of sensor NLR immune receptors and overexpression of MPSR1/SNIPER2 affects flowering in LDs as well as leaf morphology^21^. Here, we show that the effect of *mpsr-ssm136* on flowering is much stronger in the *srr1-1* background, suggesting that SRR1 has a role in the regulation of flowering via MPSR1 (Fig. 2).

The induced mutation in *ssm136* is located in the RING domain of the protein, and likely leads to changed efficiency of ubiquitination of target proteins. This would be in line with the observation of Kim et al. that an amino acid exchange in the RING domain eliminated E3 ligase activity and led to a stabilization of its protein targets^20,26^.

To test whether MPSR1 and SRR1 physically interact, we performed Co-IP in *N. benthamiana*. Interestingly, we could only detect transiently expressed MPSR1 when the RING domain was removed, suggesting that wild type MPSR1 undergoes self-ubiquitination followed by degradation. This is in line with an in vitro assay for MPSR1/SNIPER2 expressed in *E. coli* and its homolog SNIPER1^21^. Reciprocal Co-IP experiments with MPSR1ΔRING showed that SRR1 and MPSR1 directly interact, and that this interaction does not require the RING domain (Fig. 3). This is in line with reported findings that substrate specificity of E3 ligases is not established via the RING domain but instead requires highly diverse motifs recognizing a suite of features like degron sequences, posttranslational modifications or secondary metabolites^33^. A similar mechanism was observed in F-box type E3 ligases, where the F-box domain recruits the protein degradation machinery, while a separate protein-protein interaction domain specifically binds to the targets for degradation^34^. Therefore, SRR1 may either be directly targeted for degradation by MPSR1 or might act as an indirect factor that is not ubiquitinylated and guides MPSR1 to other flowering-related targets instead. Our in vivo degradation assay provides a first qualitative insight that SRR1 may indeed be a direct target of MPSR1, as we could detect a degradation product of SRR1-GFP upon co-expression with MPSR1 in the presence of the proteasome inhibitor MG-132 that was absent upon co-expression of SRR1-GFP and MPSR1ΔRING (Fig. 3c). No smaller degradation products were detected, suggesting that the observed 35 kDa product is either stable, or that the remaining protein is entirely degraded. However, abundance of total SRR1-GFP did not noticeably increase upon MG-132 treatment in samples with co-infiltrated FLAG-MPSR1, indicating that only a small portion of the SRR1-GFP substrate is prone to regulation. This would also be in line with the low amount of FLAG-MPSR1 found in this sample, owing to the rapid self-ubiquitination that might need dedicated conditions to inhibit proteasomal decay. Additionally, indirect effects through components of the *N. benthamiana* ubiquitination system may also be possible.

The *MPSR1*_*promoter*_::*LUC* reporter assay revealed that *MPSR1* is light-repressed, rapidly induced after dusk, and gradually declines throughout the night in SDs (Fig. 4h). This expression pattern mirrors that of winter photoperiodic genes such as *PHLOEM PROTEIN 2-A13* (*PP2-A13*)^30^, positioning *MPSR1* as a potential new marker gene for winter photoperiodic genes. The expression of *PP2-A13*, along with other photoperiodic genes like *MYO-INOSITOL 3-PHOSPHATE SYNTHASE 1*, is regulated by the metabolic day length measurement (MDLM) system which relies on the circadian clock and photosynthesis to support rosette fresh weight accumulation in LD and SD conditions^35–37^. It remains to be determined whether the expression of *MPSR1* is also regulated by the MDLM system. MDLM winter genes are regulated by photosynthesis and photosynthesis-derived sugars, possibly involving the AP2/EBF *cis*-element, which may be crucial for post-dusk phasing in SDs^36^. Future studies should explore whether *MPSR1* is a MDLM winter gene. Such investigations would provide valuable insights into the functions of MPSR1 and expand our understanding of the MDLM system.

In summary, we identified the RING-type E3 ubiquitin ligase MPSR1 as a SD-induced regulator of flowering. The floral repressive effect of the *mpsr1-ssm* allele identified from our suppressor screen was stronger in *srr1-1* than in wt, indicating that SRR1 might work to attenuate MPSR1 function. As we previously identified SRR1 as a focal point regulating several flowering pathways under non-inductive conditions^6^, exploring the MPSR1 substrates could be the next step to understand how SRR1 regulates flowering time.

## Materials and methods

### Plant material and growth conditions

All Arabidopsis plant lines used in this study are derived from common laboratory strains. No seeds or plant material was collected from the wild and all methods involving plants were carried out according to institutional, national, and international guidelines and legislation.

The T-DNA mutant *srr1-1* in the Col-7 background has been described^6,7^. The *mpsr1* mutant (SALK_135453) was obtained from ABRC and has been described before^21^. *CCA1*_*promoter*_::*LUC* was described previously^31^. All seeds were stratified for 2–3 d at 4°C before grown on soil. Seeds were surface sterilized and stratified for 3 d at 4 C before plating on agar-solidified half-strength MS (Murashige & Skoog) medium (Duchefa, Haarlem, Netherlands) supplemented with 0.5% sucrose and 0.5 g/L MES. Plants were grown in Percival incubators AR66-L3 (CLF Plant Climatics, Wertingen, Germany) in 100 µmol m^− 2^ s^− 1^ light intensity, with the light-dark and temperature conditions as indicated.

For the second-site suppressor screen, ca. 20.000 *srr1-1* seeds were mutagenized overnight in sodium phosphate buffer (pH 7.5)^38^. Subsequently, 0.3% ethylmethanesulfonate (Sigma Aldrich/Merck, Darmstadt, Germany) was added and the mixture was incubated for 15 h with rotation. Seeds were then washed 20x with water and distributed on soil in 280 batches. After 3 days of stratification at 4°C, the seeds were transferred to LD growth conditions. M1 seeds were harvested in batches and 100 M2 seeds each from 11 randomly selected batches were used for the initial screen.

### Flowering time

Seeds were germinated as described above and grown on soil in a random fashion. Flowering time was determined by counting the rosette leaves when the bolt was >0.5 cm tall^39^.

### DNA extraction for sequencing

Leaf material was sampled from 29 individuals in a segregating *ssm136 x srr1-1* BC1F2 population with at least 25% more leaves at bolting compared to *srr1-1* control plants, frozen in liquid N_2_ and ground to a fine powder. Equal amounts of each plant sample were pooled and this pool was extracted using a DNeasy Plant Maxi Kit (Qiagen, Hilden, Germany) according to the manufacturer’s instructions, with the following exceptions: 2x the amount of lysis buffer was used, all centrifugation steps were done for 10 min, and the empty column was centrifuged for 15 min and heated at 37°C for 10 min before elution to avoid ethanol contamination.

### Sequencing

For *ssm136*, 7 µg of DNA were used for library preparation (Novogene, Hong Kong). After quality control, libraries were constructed using the Illumina TruSeq Library Construction Kit. Pair-end sequencing was performed on Illumina^®^ HiSeq platform, with the read length of PE150 bp at each end. Base calling was done with the CASAVA software. Mapping was performed by Novogene using the BWA, SAMtools and Picard softwares, followed by SNP/Indel detection (SAMtools) and variation annotation (ANNOVAR). The same process was used to sequence a pool of *srr1-1* plants.

To create a SNP candidate index and avoid false detection of polymorphisms, all SNPs also present in *srr1-1* were filtered out, as well as low-quality and multiple-hit reads^40^. The SNP index was calculated by comparing the total number of reads to the number of reads for a non-reference base and plotted according to position on the chromosome.

### dCAPS primer design

To determine the presence of SNPs in the mutagenized plants, primers were designed to amplify the genomic region surrounding the SNP of interest. This was followed by digestion of the PCR product by restriction enzymes that only digested either the mutated product or the wt product, according to the derived Cleaved Amplified Polymorphic Sequences (dCAPS) method. Primers were designed using the dCAPS finder software, 1–2 mismatches were added to the primer to create specific restriction sites, allowing digestion of only mutant or only wt sequence^41^.

### Transcript analysis

Total RNA was extracted from plant material using the Universal RNA Purification Kit (Roboklon, Berlin, Germany) following the manufacturer’s instructions. For cDNA synthesis, 2 µg of total RNA was DNase-treated with RQ1 RNase-free DNase (Promega, Walldorf, Germany) and reverse transcribed using AMV Reverse Transcriptase (Roboklon, Berlin, Germany) according to manufacturer’s instructions. qPCR was performed with iTaq Sybr Green Supermix (Bio-Rad, Munich, Germany) according to manufacturer’s instructions. The normalized expression level was determined using the ΔCt method, with *PP2A* (AT1G13320) as a reference gene as described^42^. The primer sequences can be found in Supplementary Table S2 online.

### Cloning

For the *MPSR1* complementation construct, the genomic sequence of *MPSR1* including 0.5 kb promoter region was amplified from Col-7 DNA with primers adding EcoRI restriction sites and cloned into the binary vector pHPT1^43^. The construct was introduced into *Agrobacterium tumefaciens* GV3101 and transformed in *srr1-1* by floral dip^44^.

For *MPSR1* decoy constructs, the coding sequence of *MPSR1* was obtained by PCR using wild-type cDNA as template and inserted into pENTR/D-TOPO (Invitrogen, K240020). The N-terminal (1–336 bp) and C-terminal (469–615 bp) of *MPSR1* without RING domain were obtained individually by PCR using *pENTR-MPSR1* as template and then linked with Gibson Assembly Cloning Kit (New England Biolabs, E5510S). The generated *pENTR-MPSR1∆R* was then subcloned into pB7-HFN destination vector to obtain *35S::3xFLAG-MPSR1∆R* construct using LR recombination^45,46^. To generate the *MPSR1*_*promoter*_::*LUC* construct, a 671 bp promoter sequence upstream of the *MPSR1* coding sequence, including 5’UTR, was obtained by PCR using genomic DNA as template and inserted into pDONR207 (Invitrogen) vector and then transferred into pFLASH destination vector to drive the luciferase^32^. The primers used for cloning are listed in Supplementary Table S2 online.

### Transcript analysis based on the diurnal database

Circadian transcript profiles were retrieved from the Diurnal database (http://diurnal.mocklerlab.org)^27^. Circadian gene expression time course data were from the following datasets: LL_LDHC, plants entrained in 12L:12D with hot and cold temperature during day and night, respectively, followed by transfer to LL; LL12_LDHH, plants entrained in 12L:12D with constant temperature, followed by transfer to LL; LL23_LDHH, plants entrained in 12 L:12D with constant temperature, followed by transfer to LL; 16L:8D, 16 h light 8 h dark with constant temperature; 8L:16D, 8 h light 16 h dark with constant temperature.

### Luciferase imaging

Luciferase imaging and data analysis were performed as previously described^45^. Briefly, transgenic plant seeds harboring *MPSR1*_*promoter*_::*LUC* or *CCA1*_*promoter*_::*LUC* reporters were surface sterilized and sown on ½ strength MS plates without sucrose, stratified at 4°C for two days and transferred to 12L:12D cycles for 7 days at 22°C. Seedlings were transferred onto a 10 × 10 grid freshly poured 100 mm square ½ MS plates and then treated with 5 mM D-Luciferin (Cayman Chemical Company) in 0.01% Triton-x 100 and imaged with an Andor iKon-M CCD camera at 22°C in different light conditions as indicated. During the 5 min imaging time, lights were turned off and returned to the normal lighting regime 1 min after the exposure. Images were acquired each 1 h for approximately 6.5 d. The mean intensity of each seedling at each time point was calculated using ImageJ^47^. These raw values are presented or normalized to trace plots as indicated.

### Co-immunoprecipitation and in vivo degradation assay

Constructs expressing SRR1-GFP, FLAG-MPSR1ΔRING or GFP alone under control of the Cauliflower Mosaic Virus (CaMV) 35S promoter were introduced into *Agrobacterium tumefaciens* GV3101 (pMP90)^48^. Agrobacterium cultures were grown to an OD600 of 1.0 and independently centrifuged at 4000 g for 15 min, resuspended in infiltration media (10 mM MES-KOH pH 5.7, 10 mM MgCl_2_, 150 µM acetosyringone) and adjusted to an OD600 of 0.5^49^. Cultures were mixed with an equal volume of agrobacteria expressing the viral silencing suppressor P19 from tomato bushy stunt virus and incubated at 4°C for 1 h. The third leaf from five individual plants (8–10 leaf stage, ca. 15 cm high) was mechanically infused by pressing the tip of the syringe against the abaxial surface of the leaf and applying gentle pressure to the plunger. Two days after infiltration leaves were harvested and pooled, quick-frozen in liquid N_2_, and ground to a fine powder. Protein extracts were prepared in native extraction buffer (50 mM Tris-HCl pH 7.5, 100 mM NaCl, 10% (v/v) glycerol, 1 mM DTT) and GFP-tagged proteins were pulled down using GFP Trap beads (ChromoTek&Proteintech Germany, Planegg-Martinsried, Germany). After four washing steps with IP wash-buffer (50 mM Tris-HCl pH 7.5, 100 mM NaCl, 10% (v/v) glycerol, 1 mM DTT, 0.05% (v/v) Triton X-100), the beads were boiled in Laemmli buffer and directly loaded onto SDS-PAGE gels together with fractions from the input and the supernatant of the beads after the immunoprecipitation. Co-immunoprecipitated FLAG-MPSR1ΔRING was detected with an α-FLAG antibody (Sigma monoclonal anti-FLAG M2-antibody F3165, Merck Darmstadt, Germany). Reciprocally, FLAG-MPSR1-*∆RING* was pulled down with α-FLAG beads (ChromoTek&Proteintech, Planegg-Martinsried, Germany) and co-immunoprecipitated SRR1 was detected with an α-GFP antibody conjugated to horseradish peroxidase (Miltenyi Biotec, Bergisch Gladbach, Germany). Amidoblack staining of the membrane served as loading control.

For the in vivo degradation assay, agrobacteria containing constructs expressing SRR1-GFP, FLAG-MPSR1, FLAG-MPSR1ΔRING or GFP alone under control of the CaMV 35S promoter were infiltrated into *N. benthamiana* as described above. After 1 day of expression, a 50 µM solution of MG-132 (Merck, Darmstadt, Germany) in infiltration medium was syringe-infiltrated into the previously infiltrated leaves and samples were harvested on the next day. Infiltration of DMSO in infiltration medium served as mock control. Protein extracts were prepared in denaturing buffer (62.5 mM Tris-HCl pH 6.8, 2% (w/v) SDS, 20% (v/v) glycerol, 1% (v/v) β-mercaptoethanol, 1x cOmplete Protease Inhibitor Cocktail (Merck, Darmstadt, Germany)). 30 µg of total protein per sample was loaded onto SDS-PAGE gels and blotted onto a PVDF membrane. SRR1-GFP and GFP was detected with a monoclonal α-GFP antibody (Roche 11814460001, Merck, Darmstadt, Germany). FLAG-MPSR1 and FLAG-MPSR1ΔRING was detected with the Sigma α-FLAG antibody. As secondary antibody, α-mouse IgG coupled with horseradish peroxidase (Sigma A0168, Merck, Darmstadt, Germany) was employed. Images were visualized with a cooled CCD-camera system (Fusion FX6 EDGE, Vilber Lourmat, Eberhardzell, Germany).

## Supplementary Information

Below is the link to the electronic supplementary material.

Supplementary Material 1

Supplementary Material 2

## Data Availability

Data supporting the findings of this work are available within the paper and its Supplementary Information file. Sequencing data generated for *ssm136* are accessible at NCBI´s Sequence Read Archive (SRA) via the accession number SRR30542762, bioproject PRJNA902531.
