# Supplementary material for: A genetic screen identifies the E3 ubiquitin ligase MPSR1 as a suppressor of early flowering of the sensitivity to red light reduced 1 mutant
Source: Sci Rep. 2025 Nov 21;15:41169. doi: 10.1038/s41598-025-26769-5 (PMC12639119; doi:10.1038/s41598-025-26769-5)
Supplement: Supplementary file 2 — Supplementary Material 2 [file 41598_2025_26769_MOESM2_ESM.pdf]

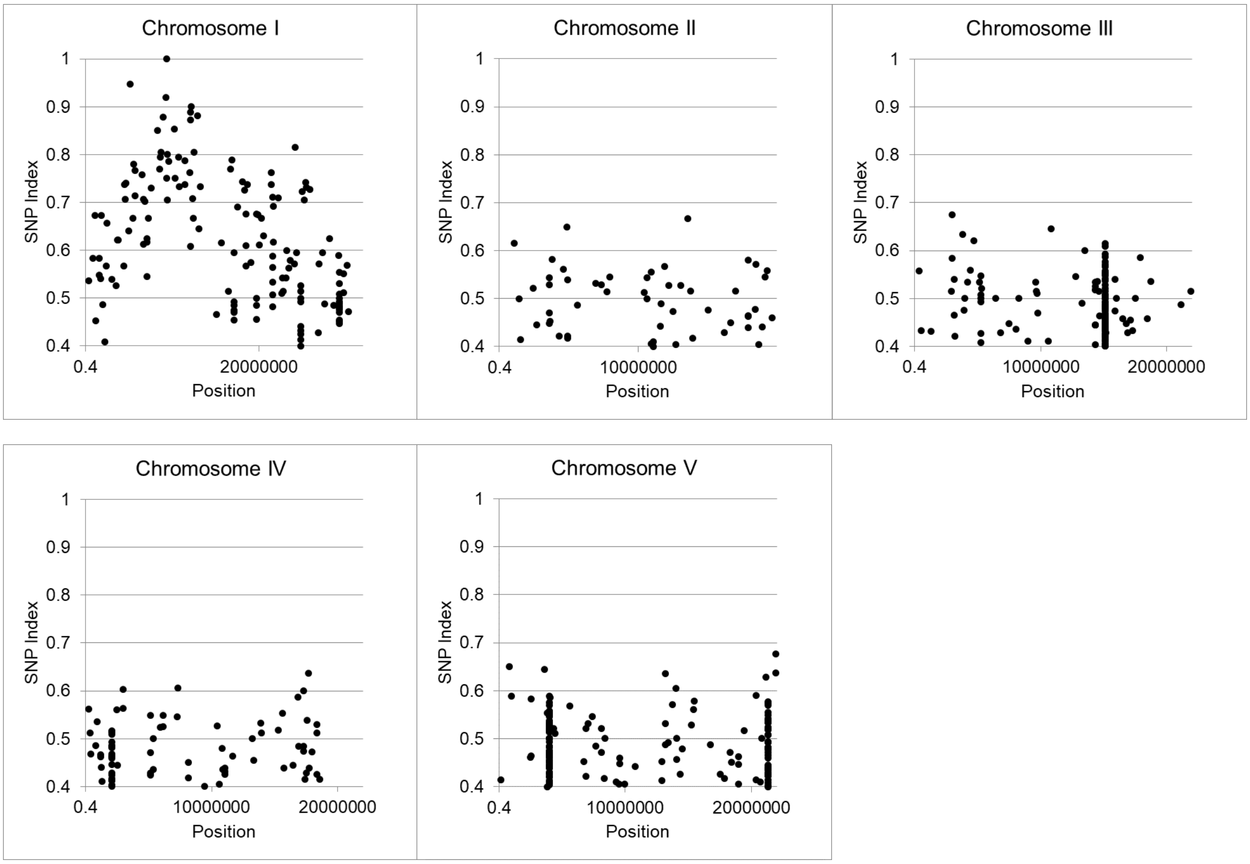

Supplementary Fig. S1: SNP index of SNPs identified in bulked *ssm136* plants plotted against genomic location for each of the five chromosomes.

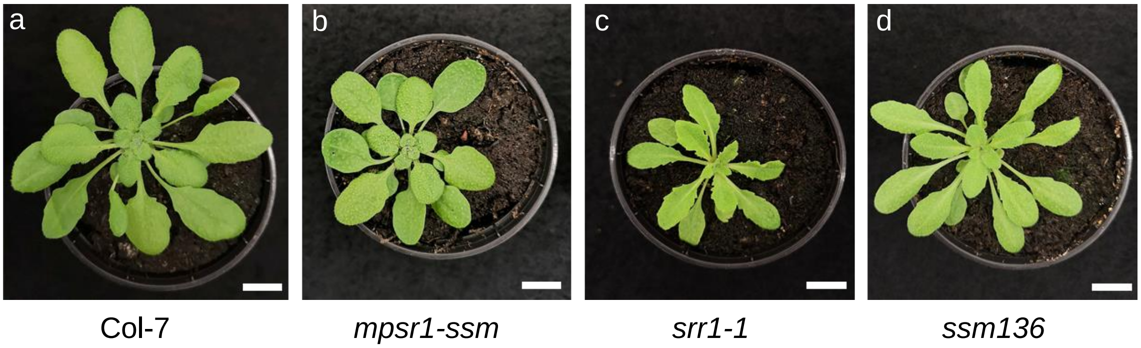

Supplementary Fig. S2: Phenotypic comparison of SD grown plants
Representative top-view images of Col-7 (a), *mpsr1-ssm* (b), *srr1-1* (c) and *ssm136* (d) plants grown on soil in SDs at 20°C. Images were taken four weeks after germination. Scale bars = 1 cm.

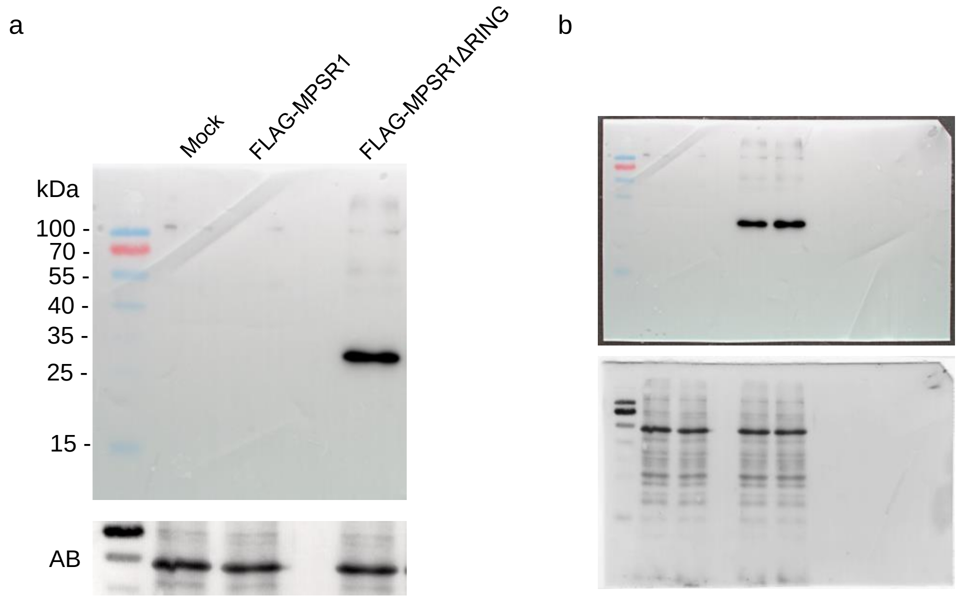

Supplementary Fig. S3:Transient expression of FLAG-MPSR1 and FLAG-MPSR1ΔRING in *N. benthamiana*
(a) Agrobacteria containing FLAG-MPSR1 or FLAG-MPSR1ΔRING constructs were infiltrated *in N. benthamiana* and leaves were harvested 2 days after infiltration. Protein expression was detected with α-FLAG antibody. Infiltration of agrobacteria without plasmid served as mock control. Amidoblack (AB) staining of the membrane served as loading control.
(b) Uncropped images of the blot shown in (a).

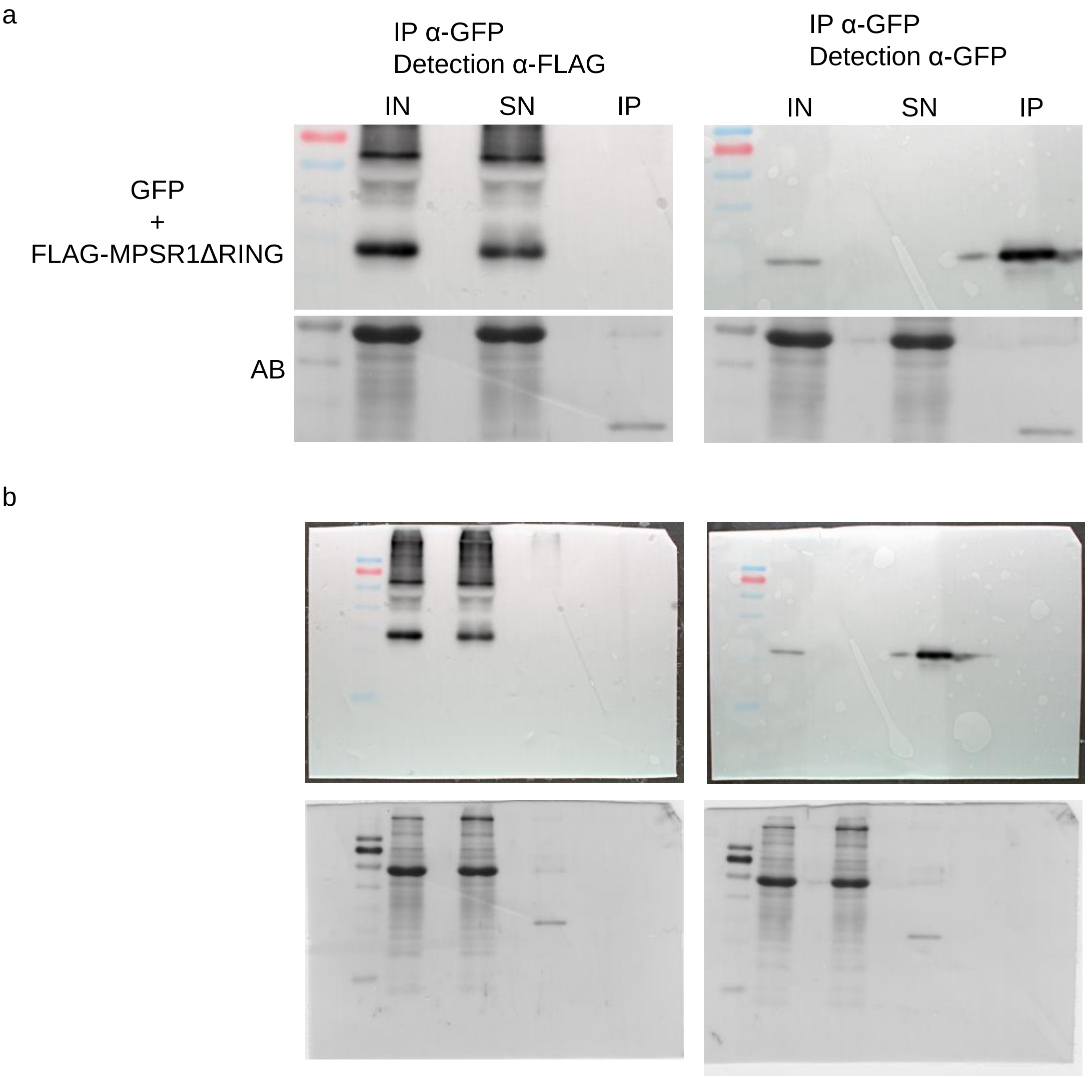

Supplementary Fig. S4: Mock Co-IP of 35S:GFP and 35S:FLAG-MPSR1ΔRING
(a) 35S:GFP and FLAG-MPSRΔRING was co-infiltrated in N. benthamiana leaves. After 2 days native protein extracts were subjected to immunoprecipitation with α-GFP beads.
Mebranes were detected with α-FLAG and α-GFP antibodies, respectively (IN, Input; SN, supernatant; IP, immunoprecipitation). Amidoblack (AB) served as loading control.
(b) Uncropped images of blots shown in (a).

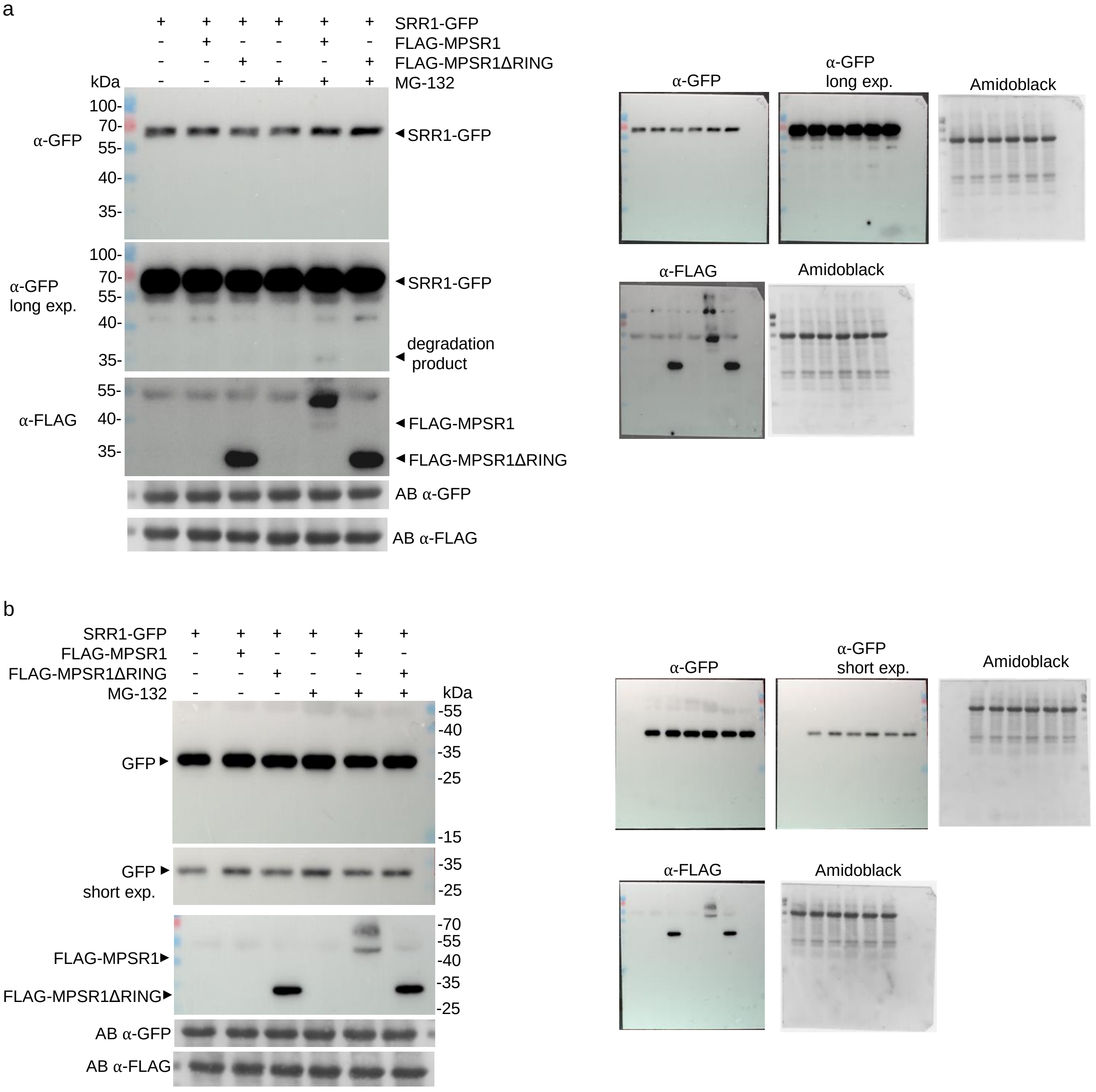

Supplementary Fig. S5: ***In vivo*** **degradation assay of SRR1-GFP and GFP**

(a) Agrobacteria carrying 35S:FLAG-MPSR1 or 35S:FLAG-MPSR1ΔRING and 35S:SRR1-GFP were co-infiltrated into *N. benthamiana* leaves. After 1 day, half of the plants were infiltrated with 50 µM MG-132 in DMSO while the other half was infiltrated with DMSO alone as mock control. Leaves were harvested after 2 days. 30 µg of denaturing protein extratcts were loaded on SDS-gels and detected with α-GFP and α-FLAG antibodies, respectively. For detection of the SRR1-GFP degradation product, a longer exposure of the α-GFP blot is given. (b) Agrobacteria carrying 35S:FLAG-MPSR1 or 35S:FLAG-MPSR1ΔRING and 35S:GFP were co-infiltrated into *N. benthamiana* leaves. The experimentwas performed according to (a). A shorter exposure of the α-GFP blot is given in order to assess differences in protein levels.
Amidoblack (AB) staining of the membranes served as loading control. Uncropped blots are presented next to the respective figures.

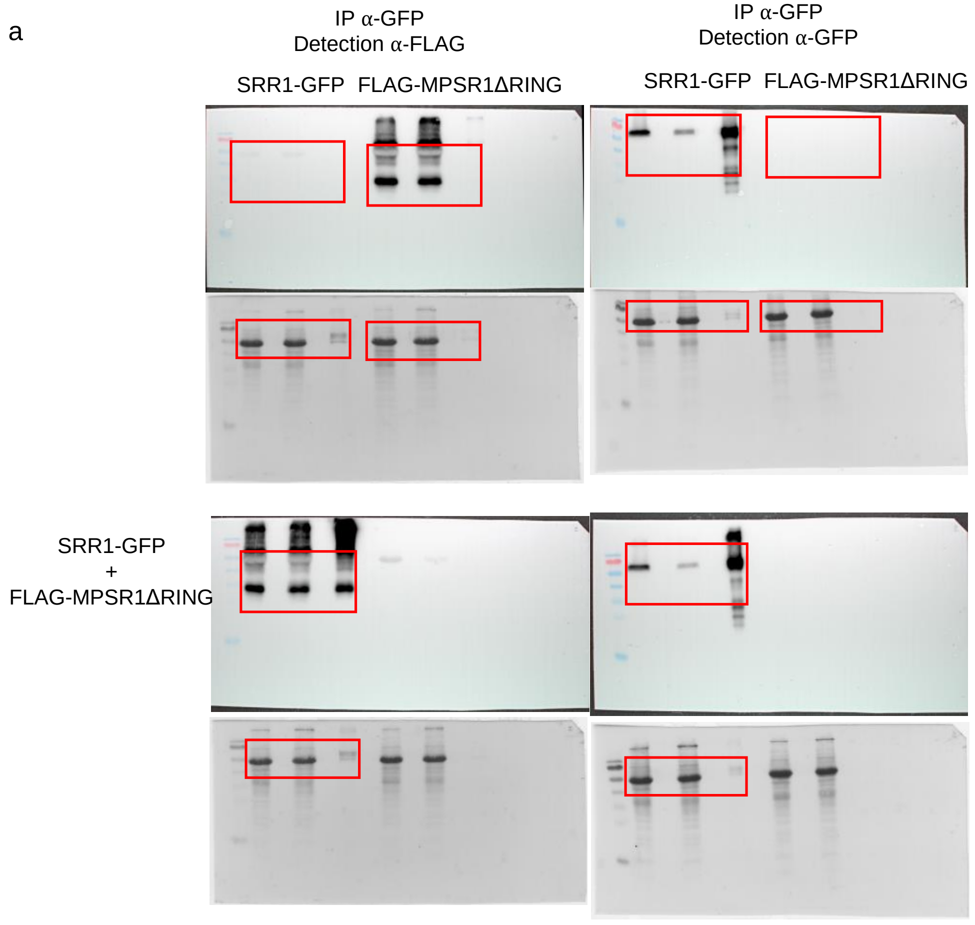

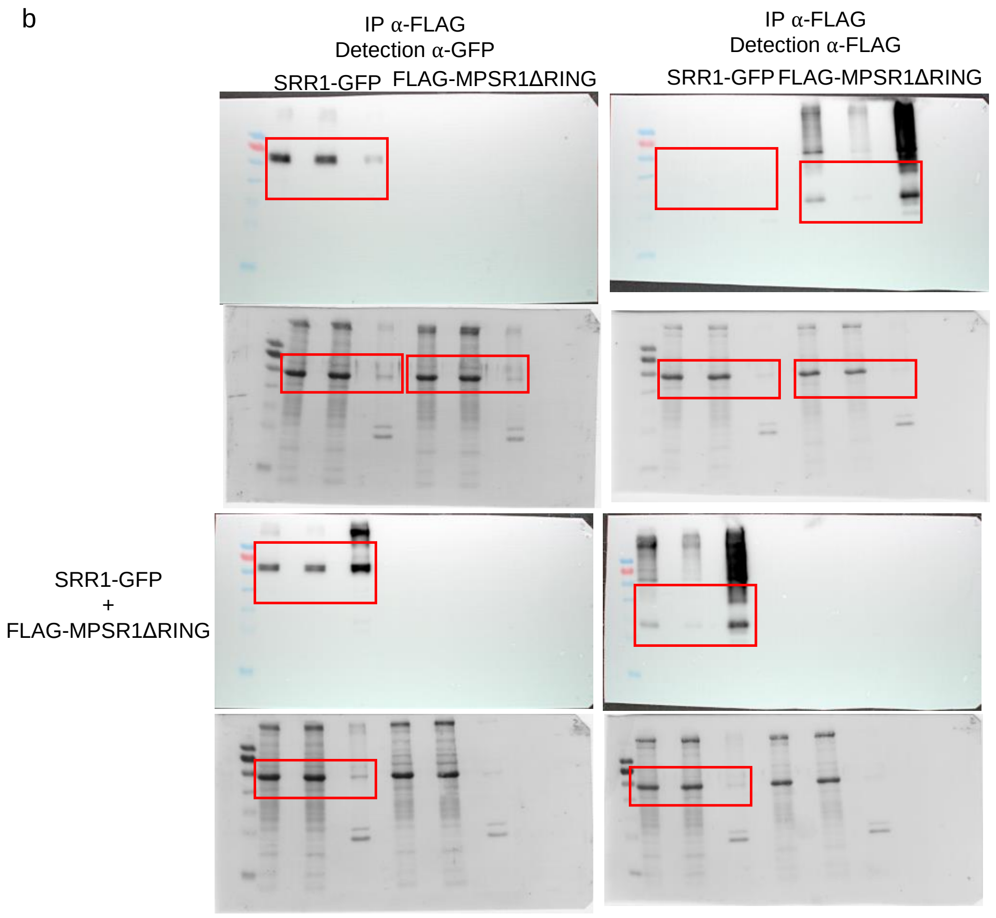

Supplementary Fig. S6: Uncropped blots corresponding to Fig. 3a+b
Uncropped blots and amidoblack stained membranes of the immunoprecipitation with α-GFP beads (a) and with α-FLAG beads (b). Cropped areas are marked with red rectangles.

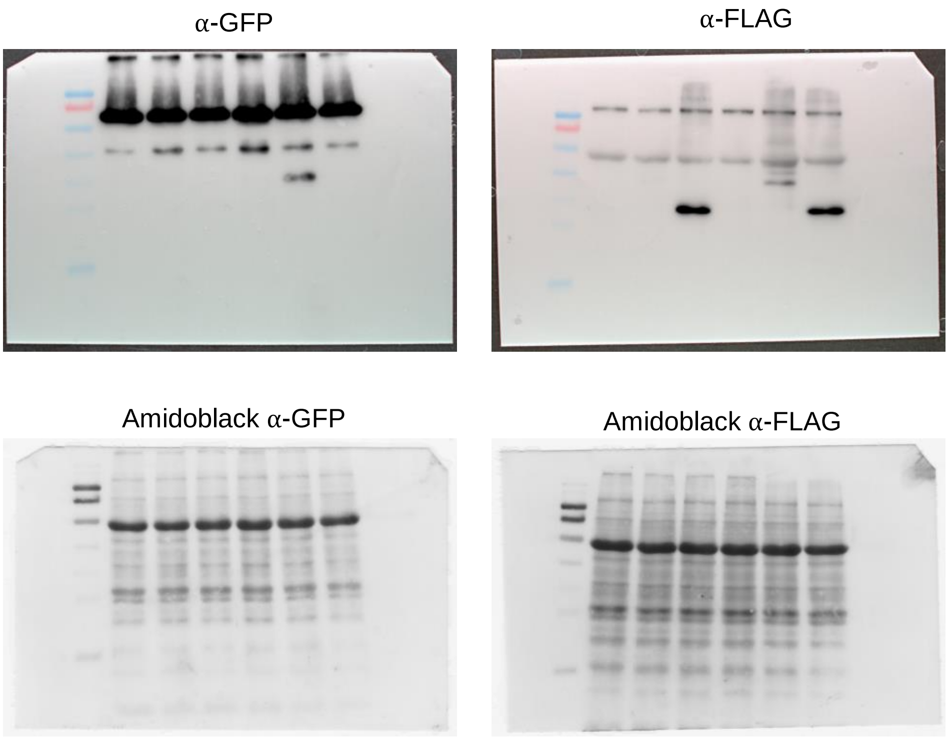

Supplementary Fig. S7: Uncropped blots corresponding to Fig. 3c
Uncropped blots and amidoblack stained membranes of the *in vivo* degradation assay.
